# A comparison and analysis of seven gun law permissiveness scales

**DOI:** 10.1186/s40621-020-00296-5

**Published:** 2021-01-18

**Authors:** Paul M. Reeping, Christopher N. Morrison, Kara E. Rudolph, Monika K. Goyal, Charles C. Branas

**Affiliations:** 1 Department of Epidemiology, Columbia University, Mailman School of Public Health, 722 West 168th Street, New York, NY 10032 USA; 2 Department of Pediatrics, Division of Emergency Medicine, Children’s National Health System, Washington, D.C USA

**Keywords:** Gun violence, Policy analysis, Gun policy, Firearms

## Abstract

**Background:**

Due to the differences in the way gun law permissiveness scales were created and speculation about the politically motivated underpinnings of the various scales, there have been questions about their reliability.

**Methods:**

We compared seven gun law permissiveness scales, varying by type and sources, for an enhanced understanding of the extent to which choice of a gun law permissiveness scale could affect studies related to gun violence outcomes in the United States. Specifically, we evaluated seven different scales: two rankings, two counts, and three scores, arising from a range of sources. We calculated Spearman correlation coefficients for each pair of scales compared. Cronbach’s standardized alpha and Guttman’s lambda were calculated to evaluate the relative reliability of the scales, and we re-calculated Cronbach’s alpha after systematically omitting each scale to assess whether the omitted scale contributed to lower internal consistency between scales. Factor analysis was used to determine single factor loadings and estimates. We also assessed associations between permissiveness of gun laws and total firearm deaths and suicides in multivariable regression analyses.

**Results:**

All pairs of scales were highly correlated (average Spearman’s correlation coefficient *r* = 0.77) and had high relative reliability (Cronbach’s alpha = 0.968, Guttman’s lambda = 0.975). All scales load onto a single factor. The choice of scale did not meaningfully change the parameter estimates for the associations between permissiveness of gun laws and gun deaths and suicides.

**Conclusion:**

Gun law permissiveness scales are highly correlated despite any perceived political agenda, and the choice of gun law permissiveness scale has little effect on study conclusions related to gun violence outcomes.

## Background

More than 32,000 people die and another 67,000 people are injured by firearms each year in the United States (Fowler et al., 2015). Rates of firearm death and injury dramatically vary state by state. For instance, according to the Centers for Disease Control and Prevention (CDC), Mississippi, Alabama, and Alaska have an age adjusted firearms death rate approximately seven times that in Rhode Island, Hawaii, and Massachusetts (Firearm Mortality Per State, 2020). Understanding the impact of state firearm legislation on health outcomes has therefore been a topic of interest for many health policy researchers. These studies commonly employ the use of scales that rate or rank “restrictiveness” or “permissiveness” of firearm legislation at the state level. These scales are useful for several reasons: to generally understand how the landscape of laws affects gun outcomes, to adjust for confounding from permissiveness of laws when it is impractical to include all state gun laws in a single analysis, and to avoid statistical complications due to collinearity of laws. This is especially relevant when a study’s statistical power is low due to small numbers, which is often an issue when units of analyses are US states over a short period of time or when the outcome of interest is rare, like mass shootings.

There are three primary ways in which the restrictiveness of state laws is rated—rankings, law counts, and scores. Rankings involve sequential lists of states from 1 to 50 based on restrictiveness based on predetermined subjective or objective assessment. Rankings assume the equivalence of each 1-unit increase. For example, Arizona might be ranked 1 in permissiveness, and Louisiana 17, (Wood, 2019) even though there might be little discernible difference between the state laws. Similarly, scores assign numeric values or letter grades based upon on predetermined criteria but allow ties. The gradation for scores can vary greatly—for example, a numeric value between 1 and 100 with decimal places—meaning that the interpretation of the results using these scales must be performed with care. Finally, law counts are the sum of active laws in each state, which allows for ties and skewed distributions, but has the disadvantage that laws are assumed to have equal effect sizes.

Permissiveness scales also arise from a variety of sources. One of the most commonly used scales (Siegel & Boine, 2019) is from the Giffords Law Center to Prevent Gun Violence’s Annual Scorecard/Legal Community Against Violence, an organization that is inherently gun-safety oriented (Gun Violence Statistics, 2019). Contrastingly, one study has recently used The Traveler’s Guide to the Firearm Laws of the Fifty States, a reference guide written for gun owners traveling across the United States that scores states on their permissiveness of gun laws (Reeping et al., 2019). Due to the differences in the ways each scale was created and the authors’ apparent political agenda, there have been questions from both the scientific and lay community about their reliability. For example, the study that used that Traveler’s Guide to the Firearm Laws of the United States was criticized by other gun violence researchers because “the gun law permissiveness scale used in the study has not been fully described, evaluated, or validated”(Webster et al., 2020) and was also criticized by the media for similar reasons (Hawkins, 2019). Furthermore, criticisms of Giffords Scorecard include articles written by pro-gun media such as, “Latest Gun Death Scorecard From Giffords Is Grossly Misleading” (Adelmann, 2019) and “Debunking Anti-Gun Giffords Law Center’s ‘Gun Law Scorecard.’“ (Tuohy, 2018).

The aim of this paper is to compare seven commonly available and prominent gun law permissiveness scales, of varying types and sources, to better understand the extent to which the choice of gun law permissiveness scale could affect the results of analyses on gun violence related outcomes. Two rankings, two counts, and three scoring scales are evaluated in this paper, arising from three gun-safety leaning sources and three gun-rights leaning sources.

## Methods

### Data sources and variables

We included the scales from the following sources for this evaluation: The Cato Institute (score), (Freedom in the 50 States, 2020) Everytown for Gun Research (count), (Gun Law Navigator, 2020) Giffords Law Center (rank and score), (Annual Gun Law Scorecard, 2020) Guns and Ammo Magazine (rank), (Wood, 2019) Siegel count of provisions (count), (McClenathan & Pahn, 2016) and the Traveler’s Guide to the Firearm Laws of the Fifty States (score) (Kappas, 2019). Giffords, Everytown, and the Siegel count are gun-safety oriented, while Cato, Guns and Ammo, and the Traveler’s Guide are gun-rights oriented. The years in which the restrictiveness scales are publicly available for use, the methods and criteria that were used to create the scale, and the location where the scales can be accessed are provided in Table 1**.** Because 2016 was the last time that data were available across all of these scales (late-July 2015 for Guns and Ammo, as 2016 is missing), we conducted our comparisons of the scales using 2016 as a reference year.

**Table 1:** Description of Scales

*Source*	*Years Available*	*Type*	*Criteria*	*Source:*
Cato Institute	2000–2016	Score	The most significant variable in the gun rights category is the concealed carry index, which takes into account shall-issue versus may-issue, carry in vehicles, local preemption, and the scope of places where concealed carry is allowed (2.2% of the freedom index). Concealed-carry permit cost (0.5% of the index) comes next. The existence of a local gun ban is worth 0.4%. 0.4% of the index is for owner licensing requirements and waiting periods on firearms purchases. At 0.2% of the index is the term of carry permits. Other variables included in this category, and worth far less than those discussed in the previous paragraph, were also included.	https://www.freedominthe50states.org/guns
Everytown for Gun Research	1991–2020	Count of Laws	There are 67 key laws that Everytown has included in the Gun Law Navigator, from the following categories: background checks, criminals, domestic violence, drugs and alcohol, mental illness, minimum age requirements, permitting process, and other.	https://everytownresearch.org/navigator/country.html
Giffords Score Card	2010–2020	Score and Ranking	“The attorneys at Giffords Law Center spend the year tracking and analyzing gun legislation in all 50 states, evaluating bills for their relative strength or weakness. Taking note of newly enacted laws, we use an exhaustive quantitative rubric to score each state on its gun law strength, adding points for safety regulations like universal background checks and extreme risk protection orders and subtracting points for reckless policies like “Stand Your Ground” and permitless carry laws. We then rank the states, convert point totals to letter grades, and compare our findings to the most recent gun death rates released by the CDC.”	https://lawcenter.giffords.org/scorecard/
Guns and Ammo	2012–2015, 2017–2019	Ranking	“we evaluate each state numerically in each of five categories: Right to Carry (RTC), access to “Black Rifles”, the states’ use-of-force laws (i.e., Castle Doctrine or CD), the prohibition of items regulated by the National Firearms Act (NFA) and a catch-all Miscellaneous (MISC) column. States are awarded 0–10 points in each category and ranked according to their total number of points. In the case of a tie, which is common, we dig deeper into the “intangibles” category and rank states accordingly.”	https://www.gunsandammo.com/editorial/best-gun-friendly-states- firearm-owners-2019/368270
Siegel	1991–2016	Count of Laws	“Using Thomson Reuters Westlaw data to access historical state statutes and session laws, we developed a database indicating the presence or absence of each of 133 provisions of firearm laws in each state over the 26-year period. These provisions covered 14 aspects of state policies, including regulation of the process by which firearm transfers take place, ammunition, firearm possession, firearm storage, firearm trafficking, and liability of firearm manufacturers.”	http://statefirearmlaws.org/
Traveler’s Guide to the Firearm Laws of the Fifty States	1997–2020	Score	This report is published yearly as a legal reference for gun owners traveling between states. The score ranges between 0 (completely restrictive) and 100 (completely permissive) for the firearm laws of all 50 states and has been used in previous research. The score considers many factors, including: standard firearms ownership and permit requirements; restrictions on semi-automatic firearms, large-capacity magazines, machine guns, and suppressors; state self-defense laws; laws governing concealed-, open- and vehicle-carry; duty to notify law enforcement of permit status; and laws that regulate firearms on school property, including vehicles parked on campus, at colleges, and K-12 schools.	https://www.gunlawguide.com/

### Statistical analyses

In order to visualize the relationship between each of the sources, we produced scatterplots of each pair of scales. We then calculated Spearman correlation coefficients between each of the scales.

To examine internal consistency, we calculated the Cronbach’s standardized alpha and Guttman’s lambda for the set of seven scales (Santos, 1999). We re-calculated Cronbach’s alpha after systematically omitting each scale to assess whether the omitted scale was contributing to lower internal consistency between scales. If this “dropped” Cronbach’s alpha appeared to be meaningfully closer to one than when all the scales are included, then that specific scale could be considered to be less reliable than the others. This method has previously been used to assess reliability (Rao et al., 2005).

We then used factor analysis (Rummel, 1988) to evaluate if all of the scales load onto a single factor. To do this, we produced a scree plot of the eigenvalues for each additional component with an oblique rotation (D’agostino Sr & Russell, 2005; Ledesma et al., 2015). Scales measuring the same construct should load onto a single factor (i.e. with an eigenvalue above one for the first factor). We also calculated the rotated factor loadings (eigenvectors) for each assessed scale, wherein a loading approaching one indicates the underlying scale strongly influences the factor (Dien et al., 2005).

### Regression analyses

We replicated the methods used in a paper published in 2013 (Fleegler et al., 2013) that examined the association between legislative strength of firearm laws and both total firearm related deaths and firearm suicides between 2007 and 2010, obtained from the Web-Based Injury Statistics Query (WISQUARS) (Underlying Cause of Death, 1999-2017) . This paper, by Fleegler et al., used a Poisson regression to estimate the association, and adjusted for state-level statistics that they received from the US Census, (American FactFinder, 2016) including race, sex, percent below poverty level, percent unemployment, percent with a college education, and state population density. An offset of population was utilized so that a relative rate could be calculated. We standardized each of the seven permissiveness scales, then repeated the analysis with each scale included as the exposure of interest.

### Sensitivity analyses

Fleegler et al. (2013) assessed state firearm law permissiveness using data from the Law Center to Prevent Gun Violence and the Brady Center to Prevent Gun Violence. States were grouped as quartiles. Because this data served as a pre-cursor for the Giffords Scorecard, (Gun Violence Statistics, 2019) we used quartiles of state permissiveness using the Giffords Rank scale to estimate the association between permissiveness and total firearm deaths. We then compared this analysis to the original analysis to confirm that the results and conclusions were accurately replicated.

Although there is little variation in the scales from year to year (as states typically do not make major changes to their legislation that would impact their score, rank, or count of laws), we also examined the results from years 2015 and 2017.

## Results

**Fig. 1:**
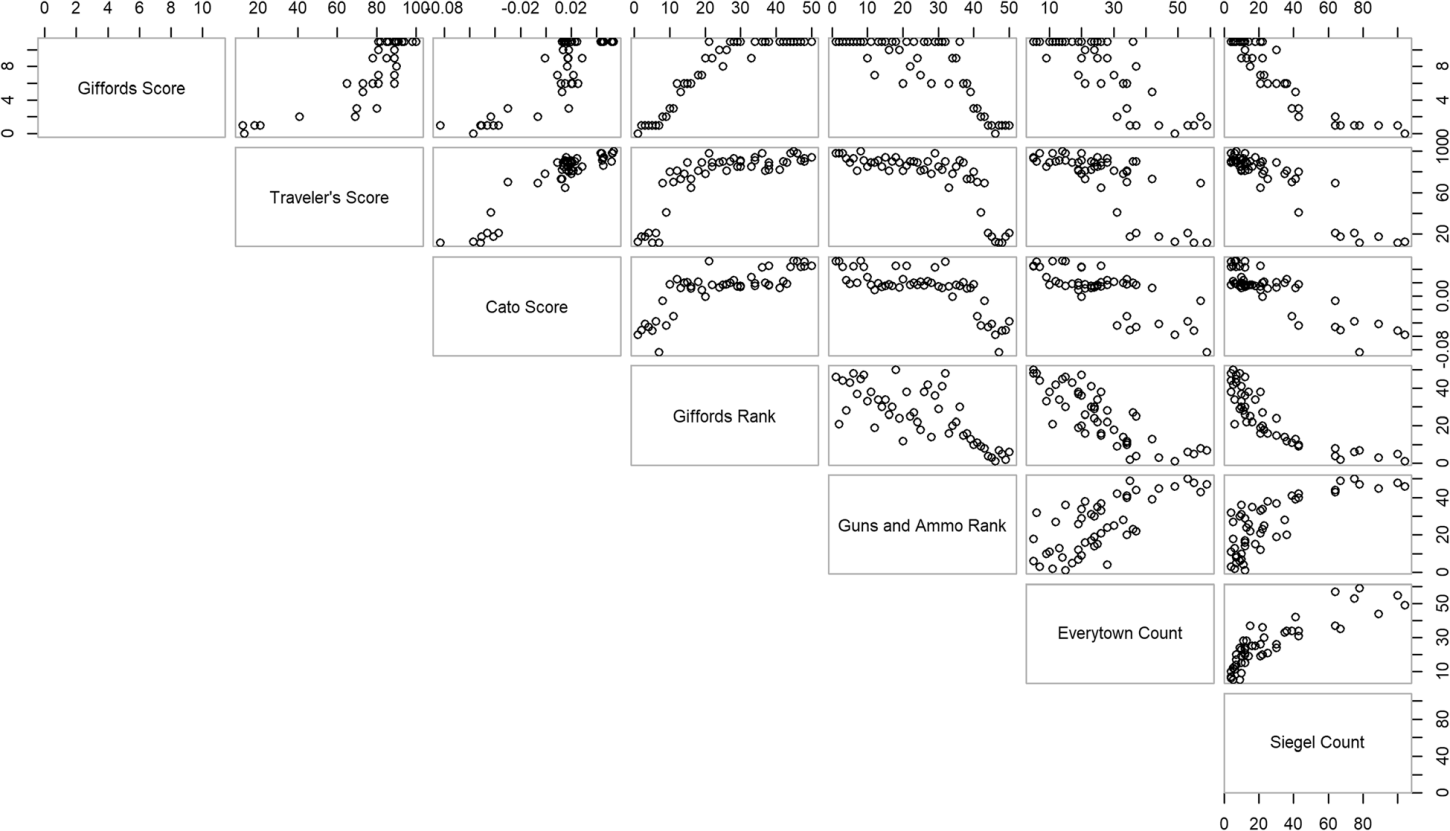
Scatterplots of each pair of scales

A scatterplot illustrating the relationships between each pair of scores is shown in Fig. 1**.** These plots demonstrate the differences between scale types. For example, the scales that are scores are more likely to have clusters of states, while rankings do not.

**Fig. 2:**
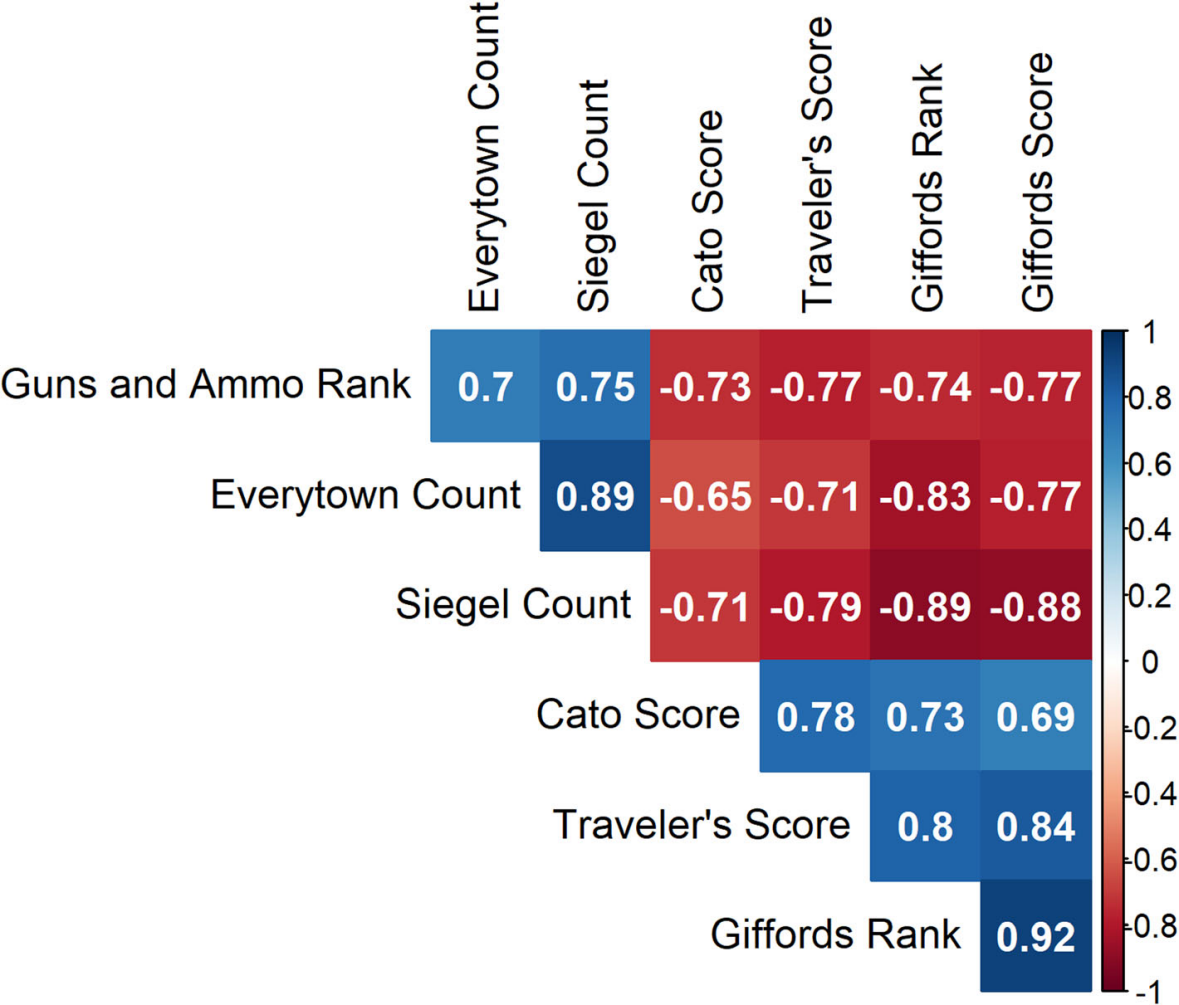
Spearman correlation coefficients between each pair of scales

The Spearman correlation coefficients between scales are shown in Fig. 2. Overall, the scores were strongly correlated with one another; the highest correlation (excluding the Giffords score vs. Gifford rank) was seen between the Everytown Count and the Siegel Count, and the Siegel Count and Giffords Rank (both at *r* = 0.89, *p* < 0.001). The scales that were the least correlated were the Cato Score and the Everytown Count (*r* = 0.65, *p* < 0.001); however, these scores would still be considered moderately to strongly correlated (Akoglu, 2018). The average correlation coefficient between scales was *r* = 0.77. Gun-rights and gun safety-oriented scores were no more or less correlated with one another. Gun-safety scores had an on average correlation coefficient of *r* = 0.86 with one another; gun-advocating sources had an average correlation coefficient of *r* = 0.75; and scores from opposite sources had an on average correlation coefficient of *r* = 0.74.

The Cronbach’s standardized alpha measuring the reliability of these scales is 0.968, and the Guttman’s lambda was 0.975, indicating that these scales were highly internally consistent. Table 2 presents the values for when a single scale is excluded from the calculation of the Cronbach’s alpha. There was no meaningful change in any of the Cronbach’s alphas, indicating again that the scores are consistently reliable.

**Table 2:** Reliability and Factor Analysis Results

	*Dropped Cronbach Alpha*	*Rotated Factor Loadings*
Cato Institute Score	0.96	0.91
Everytown Count	0.97	0.86
Giffords Rank	0.96	0.88
Giffords Score	0.96	0.95
Guns and Ammo Rank	0.97	0.82
Siegel Count	0.96	0.96
Traveler’s Guide Score	0.96	0.92

**Fig. 3:**
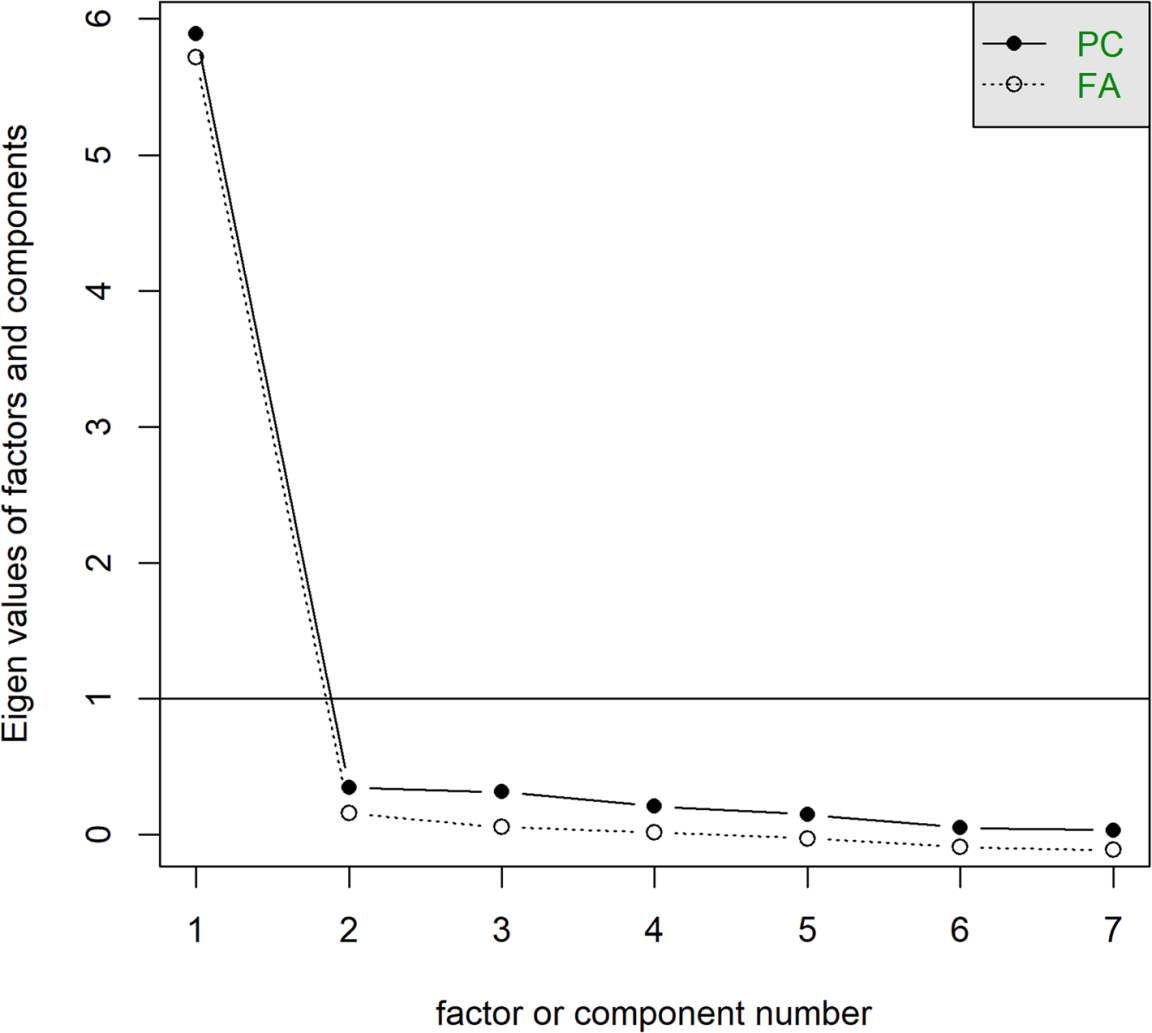
Scree Plot

A scree plot is presented in Fig. 3. The eigenvalue for the first factor was 5.72, and the second was 0.27, indicating that all scales load onto a single factor. The specific rotated factor loadings for each scale are presented in Table 2. These values ranged from 0.82 to 0.96, also indicating that these scales load heavily on a single, underlying factor.

The regression results using the methods from the Fleegler et al. paper, for both total firearm deaths and firearm suicides, are displayed in Table 3. All of the estimates for total firearm suicides are significant with similar magnitude. The lowest coefficient for the scale is 1.15 [95%CI: (1.04, 1.27)] for Giffords Rank, and the highest is 1.28 [95%CI: (1.16, 1.43)] for the Cato Score. Similar to the previous regression, all estimates for firearm suicides are significant with similar magnitude. Giffords Rank again has the lowest coefficient at 1.15 [95%CI: (1.04, 1.27)], and the Cato score is the highest at 1.32 [95%CI: (1.18, 1.46)]. The confidence intervals between all analyses are predominantly overlapping, and the conclusions would be the same for both outcomes regardless of which scale is used.

**Table 3:** Regression results for each scale

	Total Firearm Deaths		Firearm Suicides	
	*Coefficient*^a^	*CI*	*Coefficient*^a^	*CI*
Cato Score	1.28	(1.16, 1.43)	1.32	(1.18, 1.46)
Everytown Count	1.19	(1.09, 1.30)	1.25	(1.15, 1.36)
Giffords Rank	1.15	(1.04, 1.27)	1.15	(1.04, 1.27)
Giffords Score	1.24	(1.12, 1.38)	1.27	(1.14, 1.41)
Guns and Ammo Rank	1.18	(1.07, 1.29)	1.19	(1.09, 1.30)
Siegel Count	1.23	(1.13, 1.33)	1.27	(1.18, 1.38)
Traveler’s Guide Score	1.26	(1.15, 1.38)	1.27	(1.16, 1.40)

^a^adjusted for race, sex, percent below poverty level, percent unemployment, percent with a college education, and state population density

The replicated results from the regression using quartiles of the Giffords Rank scale, which are most similar to the scale used in the Fleegler et al. paper, are displayed with the results found in the aforementioned paper in Appendix Table 1**.** Although different years are examined, the coefficients and confidence intervals are nearly identical, indicating that we faithfully reproduced their methods.

All analyses produced similar findings for 2015 and 2017.

## Discussion

Through an examination of seven gun law permissiveness scales, each created using a different method (i.e. Score, Rank, and Count of Laws), we found that all of the scales are highly similar, even when comparing those more traditionally considered gun-rights vs. gun-safety scales.

Specifically, the scales are all highly correlated. We calculated measures of reliability (Cronbach’s alpha and Guttman’s lambda), and found that the scales are very reliable, which implies that one of the scales can be substituted for another. We also conducted a factor analysis, which showed that the scales all load onto a single factor. Finally, in our replication of a previous study, the choice of scale did not change the ultimate conclusion of the analysis.

There has been speculation that the different operationalizations of the scales, the different sources that the scales arise from, or the different laws that are included in the scales can result in meaningfully different conclusions. Our findings provide no evidence to support this criticism. This may be because states typically pass a series of laws that make their gun laws more restrictive or push legislation of all types that make their gun laws more permissive. Although a single law may not be included in the construction of the scales, the presence of other laws of similar type are highly indicative that the law would have been included. For example, states with stricter conceal carry requirements are also more likely to have stricter background check requires and child access prevention (CAP) laws (Gun Law Navigator, 2020).

The choice of scale for use in a given study as a composite measure of gun law strength may be immaterial. Nevertheless, practical considerations may necessitate selection of one scale over another, in part because the scales assessed here are available for different years. Ideally, a single scale would be chosen for all gun related research, as it would make it easier to compare results; otherwise, to generalize the findings to other research, the estimates would need to be standardized similarly to the way that was done here.

These scales also do not inform researchers what policies should be implemented, as they still represent a conglomerate of permissiveness, nor are they useful for regression discontinuity analysis, as there is little change from year to year in each state. Instead, these scales should be used to control for confounding by laws that are typically highly collinear. For example, an investigator might want to use the scale as a confounder in their analysis to adjust for the extent to which gun laws affect a specific relationship between an exposure and outcome. This is especially useful as there can be more than one hundred laws or policies enacted in a single state that would make individual control for each impossible due to issues of collinearity. Additionally, determining the reliability of these scales is important to address due to the criticism of past research by the academic and lay community.

A limitation of this work is that we did not include all possible scales in the analysis. We did, however, use scales that are calculated in different ways and from different sources, and have covered the most commonly used scales in firearm research to date. We also only primarily examined 2016, the most proximal year for which data from all scores were available. Regardless, we do not believe that the use of a different year would result in significantly different findings. A sensitivity analysis for 2015 and 2017 was performed (but excluded the Siegel Count and Cato Score as it was unavailable in 2017), and the results were the same across sources. Overall, the scales are relatively constant over across these years, with states like California and Massachusetts on one end of the spectrum, and Arizona and Alaska on the other end. Finally, we did not examine the extent to which the prevalence of gun ownership is correlated with the gun law permissiveness scales; however, previous research has found the two to be highly correlated,(Reeping et al., 2019) and gun ownership is likely contributing to the extent to which these scales load on a single factor.

## Conclusion

In a comparison of seven scales, created with different methods (score, rank, and count of laws), and across gun-advocating and gun-safety organizations, all of the scales are highly correlated, reliable, and load onto a single factor. We do not find evidence that conclusions from analyses on gun violence related outcomes would differ meaningfully based on choice of scale.

## Data Availability

The datasets used and/or analyzed during the current study are available from the corresponding author on reasonable request.

## Appendix

**Table 4:** Replication of Fleegler et al. results for total firearm deaths

Fleegler et al. Analysis (2007–2010)			Giffords Rank (2016)		
	*Coefficient*^a^	*CI*		*Coefficient*^a^	*CI*
1 (0–2 laws)	Ref.	Ref.	1 (x ≥ 38)^b^	Ref.	Ref.
2 (3–4 laws)	0.92	(0.74, 1.10)	2 (26 ≤ x < 38)	0.92	(0.74, 1.13)
3 (5–8 laws)	0.88	(0.65, 1.19)	3 (14 ≤ x < 26)	0.86	(0.69, 1.07)
4 (9–24 laws)	0.58	(0.37, 0.92)	4 (x < 14)	0.61	(0.45, 0.82)

^a^adjusted for race, sex, percent below poverty level, percent unemployment, percent with a college education, and state population density

^b^x is the ranking given by Giffords for each state (1–50)
